# Isolated splenic sarcoidosis: an unexpected cause for splenomegaly (a case report)

**DOI:** 10.11604/pamj.2025.52.191.50131

**Published:** 2025-12-31

**Authors:** Christoffel Roche McDuling, Ellie Georgiou, Carina Labuschagne

**Affiliations:** 1 Paarl Provincial Hospital, 10 Hospital Street, Lemoenkloof, Western Cape, South Africa,; 2 Department of Pathology, National Health Laboratory Service, University of Stellenbosch, Tygerberg Hospital, Cape Town, South Africa

**Keywords:** Tuberculosis, sarcoidosis, splenomegaly, acute care surgery, case report

## Abstract

Sarcoidosis is an idiopathic disease characterised by non-caseating granulomas affecting all organ systems. Sarcoidosis most commonly affects the lungs in >90% of cases but can present with extra-pulmonary manifestations; however, isolated splenic sarcoidosis is extremely uncommon. Isolated splenic sarcoidosis proves to be even more rare in a region where tuberculosis is endemic and presents with similar clinical manifestations, which makes diagnosis more challenging. This report describes the case of a young female presenting with non-specific abdominal complaints and clinical splenomegaly with unknown cause, and how the patient was worked up and the challenges that were faced in the diagnostic and therapeutic journey. The value of laparoscopic splenectomy is demonstrated, and the need for a combination of clinical, biochemical, radiological and histological modalities.

## Introduction

Sarcoidosis is a multi-system granulomatous condition that generally presents with lung manifestations, and patients may have a non-specific cough and chest X-ray findings [1]. The disease most commonly affects the respiratory system, but it can involve virtually any tissue type; it is rare to present with isolated splenic involvement. This unusual presentation, in combination with the fact that tuberculosis is endemic to South Africa, makes the diagnosis thereof much more challenging [2]. The prevalence of TB in South Africa in 2018 was 737 per 100,000 population, making South Africa part of the top 30 high-burden countries, contributing by itself 3% of global cases [3].

This case reports on a young female in her late twenties presenting with a history of intermittent abdominal pain. Ultrasonography revealed marked tender splenomegaly and an inhomogeneous spleen with splenic lesions. During her surgical follow-up, the patient underwent computed tomography (CT), which revealed splenomegaly with multiple cystic lesions encompassing most of the splenic parenchyma; no clear cause could be deduced from the imaging and clinical presentation alone. The patient underwent an elective, uncomplicated laparoscopic splenectomy. Postoperatively, the patient recovered well and was discharged soon after surgery. We report a case of isolated splenic sarcoidosis in a young healthy patient and the diagnostic process and difficulties faced.

## Patient and observation

**Patient information:** a female in her late twenties with no known comorbidities presented to the emergency department with a one-month history of left upper quadrant pain that was progressively worsening. She had no associated vomiting or history of trauma. Clinically, the patient had normal vital signs.

**Clinical findings:** the patient appeared to be a healthy young female, not acutely ill-looking. On focused abdominal examination, the patient was extremely tender over the epigastrium and left upper quadrant with an enlarged palpable spleen. No peritonitis, ascites or signs of portal hypertension were present.

**Timeline of current episode:** December 2024: first presentation of non-specific complaints to the emergency centre, January 2025, second presentation to the emergency room with abdominal complaints and ultrasound and CT imaging done. Patient reviewed at the surgical outpatient department. February 2025: patient underwent a laparoscopic splenectomy. March 2025: the patient was followed up at the surgical outpatient department, histology, imaging and biochemical investigations concluded the diagnosis of sarcoidosis.

**Figure 1:**
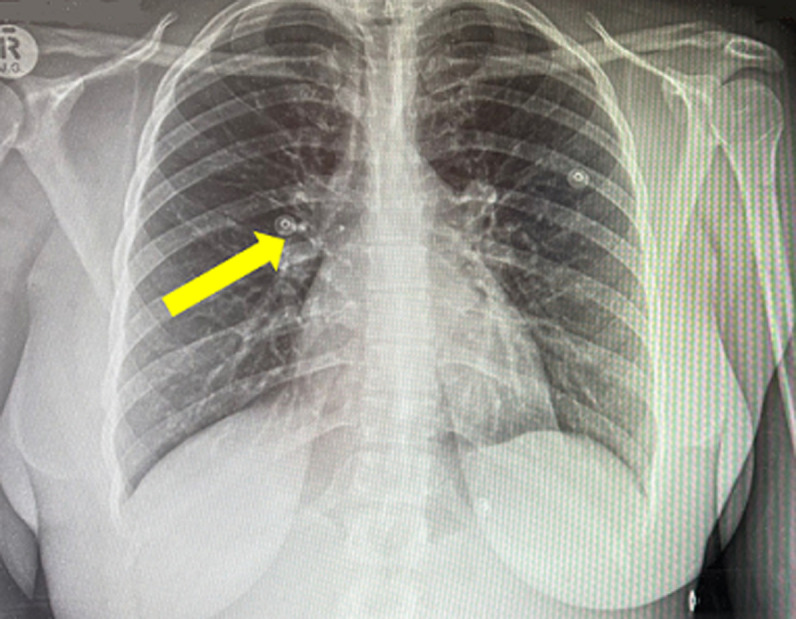
chest X-ray: demonstrating hilar lymphadenopathy (yellow arrow)

**Diagnostic assessment:** routine blood tests were done, the patient had normal electrolytes and renal functions with normal liver function tests, and lipase, but did, however, show a low Hb of 10.4 g/dL (normal range 12.0-15.0 g/dL). A plain chest radiograph showed no pneumoperitoneum, but significant hilar lymphadenopathy was seen in Figure 1. The abdominal ultrasound demonstrated a markedly tender spleen consisting of inhomogeneous echo texture with ill-defined areas of decreased echogenicity, and a round cystic component in relation to the hilum.

**Figure 2:**
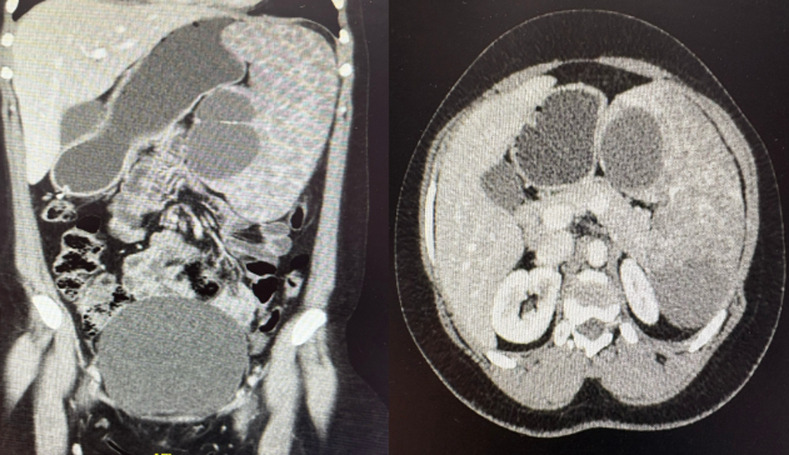
computed tomography abdominal views, left-coronal and right-transverse

Computer tomography showed an enlarged spleen with multiple cystic lesions, varying in size, involving the entire spleen, replacing normal parenchyma (Figure 2), with the largest cysts measuring 8.8 x 5.1cm and 6.1 x 3.3cm, respectively. There were no associated peri-splenic fat stranding or collections suggesting an acute infectious process; no other pathological features were noted in the rest of the abdomen. Serological work-up for echinococcus and amoebiasis was negative, along with a negative human immunodeficiency virus test. Unfortunately, the imaging studies yielded no clear or definitive diagnosis; the need for tissue for histological assessment was evident, but proved to be challenging as to how such tissue would be obtained without subjecting the patient to unnecessary risks.

**Figure 3:**
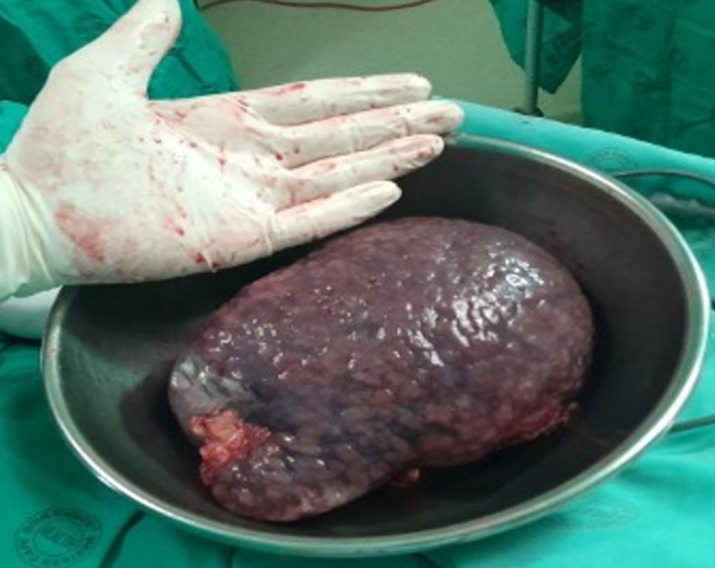
macroscopic spleen

**Therapeutic intervention:** a laparoscopic splenectomy was chosen due to the degree of discomfort the patient experienced (Figure 3) and the diagnostic uncertainty that was faced, and would possibly not just yield a diagnosis but potentially be therapeutic to the patient. The patient underwent a laparoscopic splenectomy and had an uncomplicated course postoperatively; she was discharged two days after surgery.

**Figure 4:**
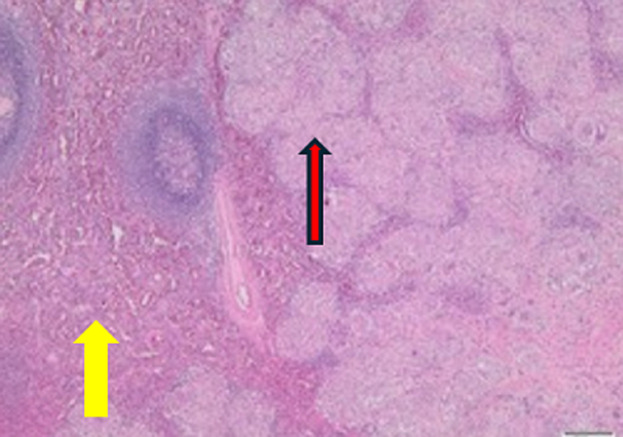
microscopic spleen: normal red pulp and white pulp of the spleen (yellow arrow) replaced by non-necrotising granulomas (red arrow)

**Figure 5:**
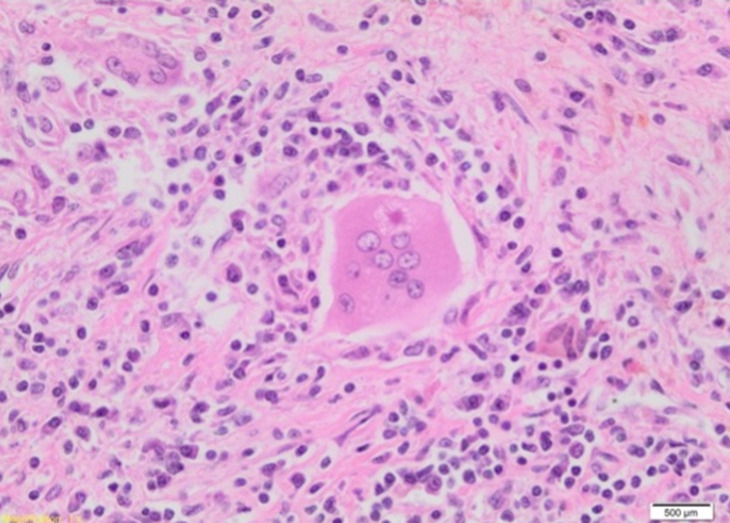
intracytoplasmic star-shaped asteroid body

**Follow-up and outcome of interventions:** the histology of the spleen showed necrotising and non-necrotising granulomas replacing most of the splenic parenchyma (Figure 4, Figure 5). Several surrounding splenic hilar lymph nodes also showed granulomatous inflammation. No signs of acute or chronic infection, including tuberculosis and fungal infection, were present. No malignancy was present in the spleen or lymph nodes. The patient was referred to the internal medicine department for further workup. The patient tested negative for tuberculosis but had elevated serum angiotensin converting enzyme (s-ACE) levels of 74 U/L, which, combined with the hilar lymphadenopathy on chest radiography and negative tuberculosis workup, was sufficient for the diagnosis of sarcoidosis after a multidisciplinary internal medicine team was consulted. The patient did not require further treatment as she was asymptomatic after surgery, and the hilar lymphadenopathy subsided. The patient was scheduled for regular follow-up appointments with the internal medicine outpatient department.

**Patient perspective:** “I had no idea what was wrong with me and started to get worried about cancer, I am relieved that I finally have an answer. The process has been stressful.”

**Informed consent:** this case report has been conducted with the full knowledge and permission of the patient and Paarl Provincial Hospital management. Signed documents were acquired stating the patient was fully informed of the process and granted her approval.

## Discussion

Sarcoidosis is poorly studied in Southern Africa with no formal epidemiological studies available, but prevalence is estimated to be between 3.7 and 23.2 per 100 000 patients according to a study done in the 1970's [4]. Diagnosis of sarcoidosis poses a challenge due to the clinical and radiological similarities to tuberculosis (TB). TB is extremely prevalent in South Africa. According to 2022 World Health Organisation statistics, the incidence of TB is estimated at 468 new cases per 100 000 people. Due to the high burden of disease, treatment and screening are focused on TB, whilst cases of sarcoidosis are frequently misdiagnosed and mistreated.

Sarcoidosis is a multisystem granulomatous condition of uncertain origin with the most common site of manifestation being the lungs [1]. Splenomegaly as the index presentation for sarcoidosis is a very rare occurrence; isolated splenic sarcoidosis has only been documented in as few as 6.5% of cases [1-5]. The aetiology of sarcoidosis is thought to be multifactorial, with the incidence varying worldwide. This may be attributed to differing genetic factors, HLA alleles and environmental exposures. One such exposure may be due to tuberculosis, with serum samples often containing mycobacterial antigens to Tuberculosis [1]. The prevalence of sarcoidosis in South Africa is not well studied or described, with some data estimating the number of cases between 4 and 25 per 100,000 annually [4]. Due to the high prevalence of tuberculosis in Southern Africa and the clinical and radiological similarities with sarcoidosis, the disease is frequently misdiagnosed [2].

Confirming the diagnosis of sarcoidosis remains challenging, a single confirmatory test for the disease does not yet exist and the diagnosis is based on a combination of clinical, radiological and histological findings with the hallmark presence of non-caseating epithelioid-cell granulomas [1]. Sarcoidosis of the spleen typically presents as nodular involvement of the organ or splenomegaly [6]. Imaging in sarcoidosis remains an important part of the diagnosis; plain chest radiographs most commonly illustrate central hilar lymphadenopathy, with four stages being described according to disease burden [1]. Caution should be taken to assume the diagnosis of tuberculosis if hilar lymphadenopathy occurs in the absence of peripheral infiltrates [2]. CT imaging is not advised as a screening tool for sarcoidosis when organ involvement is determined, but it may be useful where the diagnosis is unknown. CT findings of sarcoidosis are hypodense splenic nodules, which are more sensitive for demonstrating granulomas than sonography [7]. Fluorodeoxyglucose positron emission tomography scans may be useful, where sarcoidosis is strongly suspected, as a tool to identify suitable biopsy sites [1]. The use of magnetic resonance imaging can also aid in identifying splenic lesions; however, it adds no advantage over conventional CT and is less practical in resource-constrained environments [8].

Laboratory testing frequently yields little gain, with most blood parameters remaining within normal limits in sarcoidosis; however, they may present with anaemia, thrombocytopenia and neutropenia, likely due to hypersplenism. The use of s-ACE may be useful as these granulomas generate angiotensin converting enzyme, and an elevated serum count is found in two-thirds of patients with sarcoidosis. Some studies have demonstrated a positive correlation between splenic size and s-ACE levels [1-5]. Granulomas in sarcoidosis have no specialised properties to distinguish them from other granulomatous diseases, and the use of stains (Ziehl-Neelsen) to test for acid-fast bacilli and cultures for bacteria and fungi is paramount in excluding other causes [1]. The use of splenectomy in sarcoidosis may be indicated for patients who become symptomatic in terms of hypersplenism, abdominal pain and prophylactically to avoid splenic rupture [6-9]. Furthermore, splenectomy may be indicated to differentiate and/or exclude other neoplastic lesions. Laparoscopic splenectomy for surgical removal of the spleen has become the standard of care and is associated with better recovery, minimising blood loss and post-operative complications [8-9]. Extra-pulmonary sarcoidosis of the spleen remains a rare presentation in South Africa. This case highlights the difficulty of diagnosis in resource-constrained environments, where TB is the main suspect, and laparoscopic splenectomy may be a viable option where the diagnosis is unknown.

## Conclusion

Sarcoidosis is frequently mistaken for TB in South Africa and can have similar presentations, and is especially difficult to diagnose without respiratory manifestations. This case illustrates the approach followed after a patient presented to the emergency department with non-specific findings easily attributed to other causes, and ultimately required a splenectomy to finalise the diagnosis of sarcoidosis and prevent certain complications. It is therefore that clinicians should be careful not to condemn patients to a diagnosis of TB where the diagnosis thereof cannot be confirmed; a strong index of suspicion should be kept for sarcoidosis in any patient that presents with hilar lymphadenopathy. No gold standard test exists for the diagnosis of sarcoidosis; a combination of imaging, biochemical and histological tests may be required to make the diagnosis. As demonstrated in this case, a laparoscopic splenectomy may be the only option in isolated splenic sarcoidosis and yields therapeutic, prophylactic and diagnostic value.
